# The Mediation of AI Trust on AI Uncertainties and AI Competence Among Nurses: A Cross‐Sectional Study

**DOI:** 10.1111/jan.70250

**Published:** 2025-09-25

**Authors:** Xiangxia Liu, Yuxi Chen, Wenqing Guan, Pingping Jiang, Lihui Yan, Miao Fan, Qi Zhou

**Affiliations:** ^1^ PLA 89th Group Army Hospital Wei Fang City China; ^2^ Hangzhou Linping District Integrated Traditional Chinese and Western Medicine Hospital Hangzhou China; ^3^ Tianjin Tianshi College Tianjin China

**Keywords:** artificial intelligence, competence, nurse, nursing, trust, uncertainties

## Abstract

**Aim:**

This study aimed to validate the mediating role of nurses' AI trust in the relationship between AI uncertainties and AI competence.

**Design:**

A cross‐sectional study.

**Methods:**

A purposive sample of 550 registered nurses with at least 1 year of clinical experience from three tertiary and two secondary hospitals in Jinan and Hangzhou, China, was used. Data were collected using structured questionnaires assessing AI uncertainty, trust and competence. Demographic data included gender, age, education level, years of clinical experience, professional title and hospital level. Mediation analysis.

**Results:**

Most nurses were from tertiary hospitals (88.9%), held a bachelor's degree (87.6%), and had over 6 years of experience. The mediating role of AI trust between AI uncertainties and AI competence is validated. AI uncertainties affected AI trust (*B* = 0.39, *p* < 0.0001), explaining 10% of the variance. AI uncertainties and AI trust affected AI competence (*B* = 0.25 and 0.67, *p* < 0.0001), explaining 63% of the variation. AI trust's total effect was 0.51, comprising direct and indirect effects of 0.25 and 0.26, respectively.

**Conclusion:**

Hospitals can reduce uncertainty through an AI‐transparent decision‐making process, providing clinical examples of AI and training nurses to use AI, thereby increasing trust. Second, AI systems should be designed to consider nurses' psychological safety needs. Hospital administrators utilise optimised AI technology training and promotional techniques to mitigate nurses' resistance to AI and enhance their positive perceptions of AI competence through trust‐building mechanisms.

**Implications for the Profession and/or Patient Care:**

*Impact*: Enhancing nurses' AI trust can reduce uncertainty and improve their competence in clinical use. Strategies such as transparency, explainability and training programmes are crucial for improving AI implementation in healthcare.

**No Patient or Public Contribution:**

This study focused solely on clinical nurses and did not include patients or the public.

**Reporting Method:**

The study adhered to STROBE guidelines.

**Abbreviations**
AIC: AI competence
AIT: AI trust
AIU: AI uncertainties

## 1 Introduction

Artificial intelligence (AI) is increasingly recognised as a transformative technology in multiple sectors, with healthcare representing a rapidly expanding area of application (Nilsson 1996). AI systems employ computer algorithms to simulate human intelligence for data processing and autonomous decision‐making (Meskó and Topol 2023; Amann et al. 2020). These systems are being incorporated into clinical practice to improve disease diagnosis, surgical support and personalised treatment. Despite these advancements, the integration of AI into healthcare presents significant challenges (Amann et al. 2020). The lack of transparency and interpretability in AI decision‐making, often referred to as the ‘black box’ problem, generates concerns about systematic bias and the potential for incorrect clinical judgements (Hatherley 2020; Salimzadeh et al. 2024; Banerji et al. 2023). This uncertainty, termed AI uncertainty (AIU), encompasses users' cognitive doubts regarding the competence, dependability and transparency of AI in decision‐making (Li et al. 2024). AIU can diminish trust and confidence among healthcare providers, particularly nurses, and reduce their willingness to adopt AI technologies (Au Yeung et al. 2023; Liu 2021). A primary consequence of this uncertainty is the perception of AI competence (AIC), defined as the user's overall assessment of the AI's effectiveness, reliability and intelligence in clinical tasks (Shin 2021). When AI systems are perceived as opaque or inconsistent, healthcare professionals may question their competence, which can impede integration into clinical workflows.

Nurses constitute the largest group of healthcare professionals and are responsible for the majority of direct patient care (Ronquillo et al. 2021). They operate in complex, high‐pressure environments that require rapid and accurate information processing (Van Merriënboer and Sweller 2005). As a result, nurses are particularly sensitive to uncertainties associated with AI, which may undermine their trust in AI‐assisted decisions and potentially compromise patient safety (Au Yeung et al. 2023; Liu 2021). Although nurses play a critical role and are primary users of AI tools, limited research has examined their perceptions of AIU and its effects on clinical practice. Nursing education typically emphasises clinical care over technological proficiency, leading to a limited understanding of AI mechanisms and algorithmic transparency. This knowledge gap heightens concerns regarding the reliability and ethical implications of AI (Liu 2021; Carpio 2023; Durán and Jongsma 2021).

This study aims to investigate uncertainties in AI applications among nurses. It explores the relationships between AIU, AI trust (AIT) and AIC. Drawing on the Technology Acceptance Model (TAM), we propose that nurses' perceptions of AIU negatively influence their views on the utility and usability of AI. This situation, in turn, affects their willingness to apply AI (Sternad and Bobek 2013; Wu et al. 2023). We hypothesise that AIU negatively impacts nurses' trust in AI (AIT)—a belief in the system's reliability, integrity and benevolence (Madsen and Gregor 2000)—as well as their perception of AIC. We further propose that AIT has a positive influence on AIC and mediates the relationship between AIU and AIC. By examining these relationships, this research aims to establish a foundation for optimising AI design and applications to enhance support for nurses in their complex roles.

## 2 Background

The global application of AI in nursing is rapidly expanding, with AI tools already transforming various facets of healthcare (Seibert et al. 2021). Recent studies emphasise that AI's primary role is to support clinical decision‐making, predict patient deterioration and optimise medication management (Seibert et al. 2021; Chang et al. 2022; Ruksakulpiwat et al. 2025). Early warning systems powered by AI are linked to reduced in‐hospital mortality and shorter patient stays through timely interventions (Yuan et al. 2025). AI's influence extends beyond direct care, streamlining administrative workflows and using Natural Language Processing to automate clinical documentation, allowing nurses to devote more attention to patient care (Gonzalez‐Garcia et al. 2024; Hassanein et al. 2025). In nursing education, AI‐driven simulation and virtual reality offer students hands‐on, risk‐free practice, directly preparing them for the evolving clinical demands (Srinivasan et al. 2024; Buchanan et al. 2021; Hwang et al. 2024).

Despite these advancements, the adoption of AI in nursing is primarily determined by the perceptions and attitudes of nurses, who are the main end‐users of this technology. The literature indicates that nurses acknowledge AI's potential to improve clinical outcomes, increase workflow efficiency and reduce administrative tasks, frequently regarding AI as a tool that supports patient‐centred care (Sandanasamy et al. 2025; Alruwaili et al. 2024; Salem et al. 2024; Kaplan and Uçar 2025; Wang et al. 2024). However, this optimism is tempered by substantial concerns and a limited understanding of AI (Alruwaili et al. 2024; Rony et al. 2024; Tsimtsiou et al. 2014), suggesting that AIT is not inherent and may serve as a significant barrier to its adoption (Rony et al. 2024; Witkowski et al. 2024).

Persistent reservations about AI, particularly regarding algorithmic transparency, underscore a central challenge: nurses' mistrust when decisions lack clarity (Hatherley 2020; Salimzadeh et al. 2024; Banerji et al. 2023), especially in matters concerning patient safety (Ruksakulpiwat et al. 2025). This mistrust is exacerbated by limited AI literacy, as many nurses feel unprepared by their professional training to understand or confidently use AI technologies (Liu 2021; Shin 2021; Carpio 2023; Durán and Jongsma 2021; Liu et al. 2023). As a result, uncertainty about AI's competence increases, particularly in high‐pressure contexts, reinforcing the idea that building trust is pivotal to the successful integration of AI.

Furthermore, nurses express apprehension about the ethical implications of AI, including data privacy, potential biases in algorithms that could exacerbate health disparities and the fear of a ‘dehumanisation’ of care (Ruksakulpiwat et al. 2025; LaRosa and Danks 2018; Mainz 2024). There is a concern that over‐reliance on technology may diminish the essential human touch and interpersonal relationships that are central to nursing practice (LaRosa and Danks 2018; Mainz 2024). Recent studies also highlight that nurses' perceived risks about AI can influence their willingness to adopt it, and these concerns can even affect their intention to stay in their jobs (Labrague et al. 2023). The fear of job substitution or deskilling is also frequently cited, though many studies suggest AI is more likely to redefine nursing roles than replace them entirely (Kaplan and Uçar 2025; Labrague et al. 2023). The mediation of trust has also been validated in recent research, showing that AI anxiety and job substitution anxiety can be mediated by nurses' AIT, underscoring the critical role of trust in facilitating technology adoption (Li et al. 2024; Starke et al. 2022).

Ethical concerns further amplify nurses' uncertainty toward AI. Issues of data privacy, algorithmic bias and the possible ‘dehumanisation’ of care challenge trust in technology and may also affect job retention and role confidence among nurses (Ruksakulpiwat et al. 2025; LaRosa and Danks 2018; Mainz 2024; Labrague et al. 2023). Studies highlight that perceived risks, including fears of job substitution, are moderated by the level of trust nurses place in AI, making trust a crucial mediator in technology adoption (Li et al. 2024; Labrague et al. 2023; Starke et al. 2022).

In summary, existing research demonstrates a fundamental dichotomy: nurses express optimism regarding the potential of AI, yet remain limited by concerns related to transparency, education and ethical considerations (Au Yeung et al. 2023; Liu 2021). Understanding the processes by which trust in AI is established and sustained among nurses is essential for addressing these barriers (Wang et al. 2024). This gap in the literature, which has primarily focused on public or industry professionals (Li et al. 2024; Krop et al. 2024; Novozhilova et al. 2024), necessitates a systematic study of clinical nurses' specific experiences and perceptions. This manuscript addresses a critical gap by examining the factors that shape trust and perceptions of competence in clinical nursing, thereby contributing to the broader understanding of AI adoption in healthcare.

## 3 Methods

### 3.1 Study Design, Setting and Participants

This study adopted a cross‐sectional, quantitative and descriptive design. Participants were recruited from three tertiary hospitals and two secondary hospitals across Jinan and Hangzhou. In the selected hospitals that agreed to support this study, we chose general units (medical, surgical, or medical‐surgical units) and specialised units (intensive care units, perioperative units and emergency departments) to collect data on nurses using AI.

The inclusion criteria were as follows: (1) registered nurses (RNs) or clinical nurses holding valid national licensure issued by the National Health Commission of China; (2) a minimum of 1 year of clinical experience (this criterion ensures that participants have adequate exposure to clinical decision‐making and potential AI system usage, enhancing the relevance and reliability of their responses); (3) employment in healthcare institutions where AI‐assisted tools (intelligent nursing systems, AI‐assisted diagnostics, electronic health records) had been adopted; (4) prior experience using at least one AI‐related technology within the past year; and (5) voluntary participation with informed consent. The exclusion criteria comprised: (1) individuals not engaged in direct clinical care, such as nursing administrators or full‐time educators; (2) part‐time nurses or interns; (3) those who had undergone AI in clinical nursing training programmes lasting longer than 3 days (e.g., workshops, certificate courses), including institutional or online modules; self‐directed or informal learning; (4) nurses who had participated in research projects directly involving AI application in clinical decision‐making or nursing workflows; (5) nurses from institutions without AI implementation; and (6) individuals with cognitive or language limitations.

The required sample size was determined using the 10‐fold rule commonly applied in structural equation modelling (Jackson 2003; Kline 2015). A minimum sample‐to‐parameter ratio of 10:1 was maintained, necessitating at least 210 participants to ensure robust parameter estimation. Eligible nurses received email invitations containing study details, consent information and a link to the online questionnaire.

### 3.2 Measures

The survey included standardised instruments measuring AIU, AIT and AIC. Demographic characteristics were collected using a structured questionnaire covering gender, age, education level, years of clinical experience, professional title and hospital level. These variables were used to explore bivariate associations with AI‐related constructs and as potential covariates in the mediation analysis.

AIT was measured using the 8‐item Artificial Intelligence Trust Scale that Calhoun and Merritt et al. developed. Each item was rated on a 5‐point Likert scale (5–40), with higher scores indicating greater trust in AI. A sample item was: ‘I would rely on AI without hesitation.’ The original scale demonstrated strong internal consistency (Cronbach's *α* = 0.869, Composite Reliability = 0.898) (Calhoun et al. 2019; Merritt 2011). In this study, Cronbach's *α* was 0.944.

AIU was measured using the 6‐item AI Uncertainty Scale by Madsen and Gregor. The scale employed a 5‐point Likert format (5–30), with higher scores reflecting greater uncertainty toward AI. A sample item was: ‘There are many unknowns about AI’. Previous research reported strong reliability (Cronbach's *α* = 0.846, Composite Reliability = 0.883) (Madsen and Gregor 2000). In this study, Cronbach's *α* was 0.964.

AIC was measured using the 3‐item AI Competence Scale developed by Shin (2021). This 5‐point Likert scale (5–15) measured perceived competence in AI usage, with higher scores signifying greater AI proficiency. A sample item was: ‘I believe AI produces accurate output’. The original scale exhibited satisfactory reliability (Cronbach's *α* = 0.738, Composite Reliability = 0.851). In this study, Cronbach's *α* was 0.944.

### 3.3 Statistical Analysis

All statistical assumptions were assessed prior to analysis. Normality was evaluated through skewness and kurtosis values, all of which fell within the acceptable range of ±2. Homogeneity of variances was verified using Levene's test, with no significant violations detected (*p* > 0.05). Multicollinearity was assessed using tolerance values and variance inflation factors (VIF). AIU and AIC yielded tolerance values of 0.90 and VIFs of 1.11, confirming the absence of collinearity concerns (tolerance > 0.10, VIF < 2). Descriptive statistics were computed using SPSS 25.0, summarising participants' demographic characteristics and AIT, AIU and AIC distributions. Pearson's correlation analysis was performed to examine the relationships among these variables. Skewness, kurtosis and variance inflation factors were evaluated to ensure model assumptions were met. The SPSS PROCESS macro implemented least‐squares regression to test the hypothesised relationships. A mediation model (Model 4) was constructed to explore the mediation effect (Van Merriënboer and Sweller 2005). AIT served as the dependent variable, AIU as the independent variable and AIC as the mediator. Demographic variables are covariates in the mediation model to control for potential confounding effects. The bootstrap method (5000 resampling iterations) was utilised to estimate indirect effects, with statistical significance established when the 95% confidence interval did not contain zero.

### 3.4 Ethics Statement

Participation was voluntary, and strict confidentiality was upheld. Since data collection was conducted via an online survey, explicit written consent was not required. Instead, participants provided informed consent by selecting a confirmation checkbox before proceeding with the questionnaire. They were informed of their right to withdraw at any time, and all responses were anonymised. The study received ethical approval from Hangzhou Linping District Integrated Traditional Chinese and Western Medicine Hospital on March 12, 2025 (004).

## 4 Results

Of the 600 questionnaires distributed, 550 nurses provided data, with a response rate of 91.6%. The majority of nurses were in tertiary level hospitals (88.9%, *n* = 489), 26–36 years old (41.7%, *n* = 216), bachelor's degree (87.6%, *n* = 482), 6–11 years working experience (30.4%, *n* = 167), nurse practitioner in charge (50%, *n* = 275). Bivariate analyses revealed that nurses' working experience significantly influenced AIC (*p* = 0.01). Other factors, such as hospital level, professional title and education, showed no significant association (Table 1).

**TABLE 1:** Characteristics of the subjects and scores on AI competence (*N* = 550).

Variable	Categorization	Frequency	Percentage (%)	Mean	SD	*p*
Hospital level	Tertiary	489	88.9	2.23	0.8	0.61
	Second‐class	58	10.5	2.22	0.74	
	Primary	3	0.5	1.78	1.07	
Working experience (years)	< 6	81	14.7	2.27	0.91	0.01
	6–< 11	97	17.6	2.27	0.79	
	11–< 16	167	30.4	2.23	0.77	
	16–< 21	143	26	2.31	0.79	
	> 21	62	11.3	1.9	0.66	
Professional title	Nurse	77	14	2.22	0.95	0.38
	Senior nurse	166	30.2	2.32	0.76	
	Nurse practitioner in charge	275	50	2.18	0.74	
	Associate or Chief Nurse Practitioner	32	5.8	2.18	1.05	
Education	Junior college	6	1.1	2.19	0.65	0.86
	College	51	9.3	2.17	0.89	
	BD	482	87.6	2.23	0.79	
	MD or PhD	11	2	2.29	0.54	

Abbreviations: BD, Bachelor's Degree; MD, Master's or Doctoral Degree; PhD, Doctor of Philosophy.

The total mean score for AIT was 2.61 ± 0.83; AIC was 2.20 ± 0.69; AIU was 2.02 ± 0.67 (Table 2).

**TABLE 2:** The mean scores of nurses on variables (*n* = 550).

Variable	Mean	SD
AI trust	2.61	0.83
AI competence	2.20	0.69
AI uncertainties	2.23	0.80

The findings showed that there was a strong positive correlation between AIU and AIT (*r* = −0.31, *p* < 0.001), AIT and AIC (*r* = 0.768, *p* < 0.001), AIU and AIC (*r* = 0.432, *p* < 0.001) (Table 3).

**TABLE 3:** Variable relationships.

Correlation	AI trust	AI competence	AI uncertainties
AI trust	1	0.768**	−0.310**
AI competence	0.768**	1	0.432**
AI uncertainties	−0.310**	0.432**	1

** All correlations were significant, *p* < 0.001.

**FIGURE 1:**
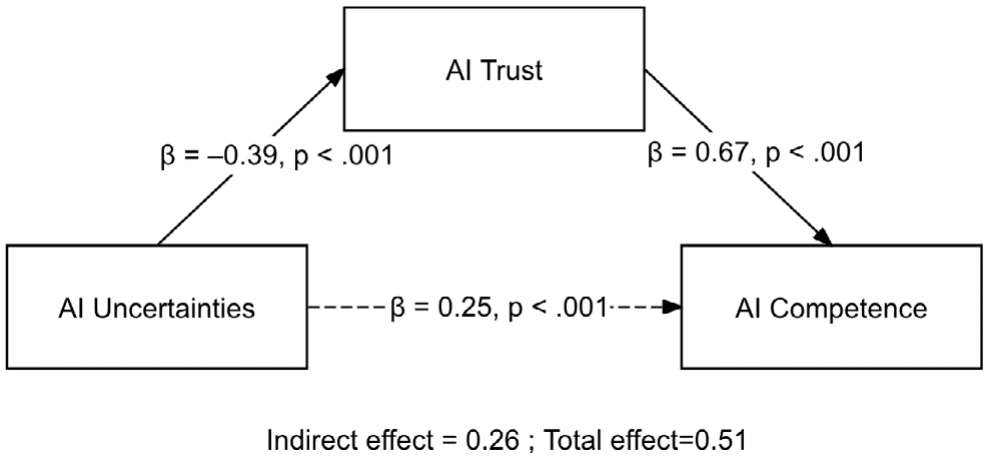
The results of mediation analysis.

The relationship between AIU and AIC mediated by the AIT was confirmed. AIU affected AIT (*B* = 0.39, *p* < 0.0001), with an explained variation of 10%. AIU and AIT affected AIC (*B* = 0.25 and 0.67, *p* < 0.0001), with an explained variation of 63%. AIT's total, direct and indirect effects were 0.51, 0.25 and 0.26, respectively. All values were significant, indicating that AIT partially mediated between AIU and AIC (Table 4, Figure 1).

**TABLE 4:** Mediation effect of AI trust between AIU and AI competence.

	AI trust				AI competence			
	Coeff.	SE	*t*	*p*	Coeff.	SE	*t*	*p*
AI uncertainties	0.39	0.05	7.64	< 0.001	0.25	0.03	7.85	< 0.001
AI trust					0.67	0.03	25.71	< 0.001
Adjust *R* ^2^	0.1				0.63			
*F*	58.31				469.02			

	Effect	BootSE	Confidence interval	
			Low bound	Upper bound
AI uncertainties → AI trust → AI competence				
Indirect effect	0.26	0.05	0.18	0.33
Direct effect	0.25	0.04	0.18	0.33
Total effect	0.51	0.05	0.42	0.6

*Note:* The working experience is controlled as a covariate.

## 5 Discussion

This study reveals the partially mediating role of AIT between AIU and AIC. This study builds on prior research, concentrating solely on public AI acceptance or willingness by investigating nurses' AIU on AIC. Additionally, it introduces the novel concept of trust as a psychomodifying factor. This study offers a novel perspective on the application of AI, specifically targeting nurses.

### 5.1 Understanding the Low Status of Nurses' AIT, Uncertainty and Competence

The nurses' AIT scores were lower than those reported by Li et al. (2024) and Calhoun et al. (2019). Primary hospital nurses scored lower than hotel staff, possibly due to the specialised nature of nursing. First, nurses often have limited exposure to AI technology and its clinical applications, leading to hesitation and diminished trust. Secondly, concerns regarding AI's reliability, privacy and ethical implications further contribute to their scepticism (LaRosa and Danks 2018; Mainz 2024). In these settings, nurses prioritise patient care, often perceiving technology integration as an additional burden rather than an asset. Therefore, this study recommends that nursing administrators strengthen educational programmes highlighting AI's practical benefits and ethical considerations (Zuchowski et al. 2024). Moreover, involving nurses in decision‐making regarding AI implementation may foster a greater sense of ownership and trust in these technologies (De Brito Duarte et al. 2023).

The nurses' AIU scores were lower than those reported by Li et al. (2024) and Lockey et al. (2020). Primary hospital nurses scored lower than technical staff, possibly due to their limited understanding of AI's technical intricacies. Nurses focus on AI's practical, patient‐centred benefits rather than its underlying design (Banerji et al. 2023). Their awareness of AI's technical limitations and uncertainties may be constrained, likely due to minimal direct interaction with the system's core mechanisms (Liu 2021).

The nurses' AIC scores were lower than those reported by Shin (2021). The findings indicate that nurses have low AIC. This situation relates to nurses' limited use of AI and lack of confidence in AI‐generated outcomes (Liu et al. 2023). Unlike skilled professionals, nurses may struggle to assess the AI's accuracy and reliability, resulting in low AIC. In addition, nursing primarily focuses on patient health. Nurses view AI as an assistive tool, which limits their capacity to make independent decisions (Kim et al. 2021). Hospitals should implement structured training programmes to enhance nurses' AIC. Nursing administrators should facilitate the active learning of AI technology among nurses, enhancing their confidence in its practical use and ultimately advancing AIC (Madsen and Gregor 2000).

AIU was negatively correlated with AIT, consistent with findings from Salimzadeh et al. (2024), Li et al. (2024), Liu (2021), Carpio (2023) and Vaccari and Chadwick (2020). There are three reasons for this phenomenon. First, information asymmetry plays a crucial role. Nurses often have limited knowledge of AI's technical foundations and operational mechanisms, leading to a diminished sense of understanding and control. According to the TAM, technology acceptance and trust depend on users' comprehension and perception. When AI systems lack transparency and precise feedback mechanisms, nurses' uncertainty increases, eroding trust (Durán and Jongsma 2021; Liu et al. 2023; Huang et al. 2024; Kerasidou et al. 2022). Second, nurses bear significant clinical responsibilities, particularly safeguarding patient privacy and ensuring healthcare security. The integration of AI raises concerns about data security and potential privacy breaches (Banerji et al. 2023; Huang et al. 2024). Insufficient transparency and regulatory oversight in AI implementation may intensify uncertainty, diminishing trust (Vaccari and Chadwick 2020; Huang et al. 2024). Therefore, hospitals should improve the transparency of AI technology by effectively communicating its features, limitations and data handling (Zuchowski et al. 2024). In addition, hospitals should strengthen their ethical review of AI systems to ensure data security and privacy protection, which can effectively alleviate nurses' concerns and enhance trust (Vaccari and Chadwick 2020).

### 5.2 The Interplay Between Nurses' AIT, Uncertainty and Competence

This study is the first to validate the coexistence of nurses' AIU and AIC. When faced with AIU, nurses must proactively improve their technological understanding to reduce uneasiness due to information asymmetry. In addition, AIC may allow nurses to acutely recognise the AIU and adopt effective coping strategies rather than avoid them or rely on external support (Liu et al. 2023). Therefore, hospitals should provide systematic support for AIC among nurses, including enhanced technical training and opportunities for clinical practice. Furthermore, explainable AI mitigates technological uncertainty, enhancing AIC and self‐confidence (Huang et al. 2024).

AIT was positively correlated with AIC, consistent with findings from Li et al. (2024), Hsieh and Lee (2024) and Jiang et al. (2023). This result may be because trust reduces nurses' psychological resistance to using AI technology, thus enhancing perceived AIC. The Technology Acceptance Model and Social Cognitive Theory suggest that individuals' AIT increases their self‐efficacy, leading them to view themselves as competent in mastering and using the technology (Durán and Jongsma 2021; Huang et al. 2024). In addition, AIT means that nurses perceive AI as reliable in clinical decision‐making, consequently enhancing their willingness to implement AI in practice. Therefore, healthcare organisations should implement strategies to enhance nurses' AIT, including optimising AI transparency, providing interpretable AI solutions and reinforcing the ethical standards of AI applications (Kerasidou et al. 2022). The design of AI systems must consider the psychological safety requirements of nurses. AI with high explainability and controllability allows nurses to trust AI despite uncertain situations.

### 5.3 Nurses' AIT as a Mediator in Uncertainty and Competence

Nurses' AIU impacts AIC and also has an indirect effect through AIT. Nurse AIT is a significant strategy for mitigating AIU and enhancing AIC. This partially mediated effect is consistent with previous theoretical studies (Li et al. 2024; Starke et al. 2022). When nurses perceive AIU, they may be sceptical about using AI. However, if they trust AI, they may be more willing to believe in AIC and use it despite existing uncertainties. Uncertainty Management Theory (UMT) posits that when confronted with technological uncertainty, individuals who trust in the reliability and controllability of technology are more likely to adapt proactively instead of avoiding it altogether (Durán and Jongsma 2021; Liu et al. 2023). Therefore, healthcare organisations must implement systematic measures to enhance AIT. These measures include improving AI transparency, optimising human‐computer interaction and strengthening ethical and privacy protections to alleviate nurses' concerns regarding the AIU (Kerasidou et al. 2022). Furthermore, through focused training and clinical practice, nurses may acquire experience in AI applications within real‐world environments, enhancing their technological adaptability and improving their AIT and AIC (Salimzadeh et al. 2024). These approaches will facilitate the effective integration and application of AI technology in clinical nursing.

### 5.4 Limitation

This study has three limitations. Firstly, the study's sample was limited to nurses in Jinan and Hangzhou, restricting the generalisability of findings. Future research should expand to diverse regions and healthcare levels to enhance applicability. Secondly, self‐reported data may be influenced by social desirability bias, affecting reliability. Qualitative methods, such as interviews or focus groups, could provide deeper insights. Finally, the cross‐sectional design limits understanding of how AIU and AIC evolve. Longitudinal studies are needed to examine their dynamic changes and long‐term impact on AIT.

## 6 Conclusion

This study reveals the partial mediating role of AIT between AIU and AIC. This study posits that the enhancement of AIC relies not solely on technology but also on the improvement of AIT. Hospitals can reduce AIU and enhance AIT by ensuring transparency in AI decision‐making, offering clinical examples of AI applications and providing training. Secondly, AI systems should be designed to consider the nurses' psychosocial safety. AI with high explainability and controllability allows nurses to trust AI despite uncertain situations. Finally, hospital administrators use optimised AI technology training and promotion strategies to reduce nurses' rejection of AI and enhance their positive perceptions of AI capabilities through trust mechanisms. Nursing administrators can implement an AI‐assisted but nurse‐led decision‐making model to enhance AIC and decrease AIU. Nurses must participate in the AI design and implementation to ensure the effective integration of AI in clinical care.

## Author Contributions

All authors contributed to the conceptualisation, data curation, formal analysis, funding acquisition, investigation, methodology, project administration, software, resources, supervision, validation, visualisation, writing – original draft, writing – review, editing.

## Disclosure

Statistics: The authors have checked to make sure that our submission conforms as applicable to the Journal's statistical guidelines described. The author(s) affirm that the methods used in the data analyses are suitably applied to their data within their study design and context and the statistical findings have been implemented and interpreted correctly. The author(s) agrees to take responsibility for ensuring that the choice of statistical approach is appropriate and is conducted and interpreted correctly as a condition to submit to the Journal.

## Ethics Statement

The Institutional Review Board of China (Hangzhou Linping District Integrated Traditional Chinese and Western Medicine Hospital) granted ethical approval. Before distributing the questionnaire, we explained the purpose of the study to the participants. We also ensured that participants were voluntary and anonymous and allowed them to opt out anytime. We stored the data securely; only research team members could access, store and process it. Hangzhou Linping District Integrated Traditional Chinese and Western Medicine Hospital approved this study on March 12, 2025 (2025‐Y‐004).

## Consent

The authors have nothing to report.

## Conflicts of Interest

The authors declare no conflicts of interest.

## Data Availability

Data will be made available on request.
